# How the Choice of Spatial Resolution Affects Freshwater Fish Species Distribution Models

**DOI:** 10.1002/ece3.73472

**Published:** 2026-04-22

**Authors:** Davide Fundaro', Jelle P. Hilbers, Koen J. J. Kuipers, Aafke M. Schipper

**Affiliations:** ^1^ Radboud Institute for Biological and Environmental Sciences (RIBES) Radboud University Nijmegen the Netherlands; ^2^ PBL Netherlands Environmental Assessment Agency Hague the Netherlands

## Abstract

Species distribution models (SDMs) are widely used to understand and predict how species respond to changes in their environment. Hence, it is critical to understand how SDMs are influenced by methodological aspects and choices. The choice of spatial resolution is an understudied subject, especially for freshwater fish SDMs. Here, we aimed to analyse how the choice of spatial resolution affects the predictive accuracy, predictor variable importance and spatial predictions of SDMs of freshwater fish species in Europe. We fitted SDMs for 49 freshwater fish species based on point occurrence records combined with environmental and anthropogenic predictor variables aggregated at five spatial resolutions as represented by nested hydrological basins (level 8 to 12 in the HydroSHEDS database). Following an ensemble modelling approach, we employed nine algorithms to establish the SDMs at each of the resolutions, using temperature, topography, streamflow, land use, human population and dam density as predictor variables. We analysed the differences in predictive performance by testing the models on a geographically independent subset of the data. Predictive performance and variable importance were highly similar across the resolutions, with a median TSS between 0.37 and 0.39 and temperature and topography being the most important variables. In contrast, predicted range size decreased towards higher resolutions, while the difference between predicted and observed range size increased. Our results indicate that the choice of spatial resolution has a small effect on the performance and predictor importance of continental freshwater fish SDMs, while significantly influencing predicted range size. The latter may have important consequences for conservation and extinction risk assessments, which often rely on estimates of range size. From a precautionary principle, establishing SDM at the highest resolution possible may help to prevent the risk of overestimating range size hence underestimating extinction risk.

## Graphical abstract

We examined the influence of spatial resolution on freshwater fish SDMs across Europe. Model performance and variable importance were consistent across scales, but predicted range size declined with finer resolution. These results highlight the importance of spatial resolution when obtaining SDM outputs for conservation purposes.

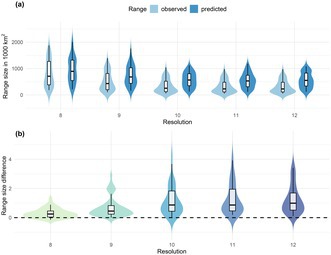

## 1 Introduction

Species distribution models (SDMs) are widely used for a variety of purposes, including understanding the habitat requirements of species, evaluating and predicting the effect of environmental changes on species distributions, or guiding conservation planning (Peterson 2006; Catullo et al. 2008; Markovic et al. 2014; Tao et al. 2023). For establishing reliable SDMs, it is key to understand their sensitivity to various methodological choices, including the selection of environmental predictors, the generation of pseudo‐absences or background data and the choice of algorithms and spatial resolution (i.e., the spatial granularity of data) (Durance et al. 2006; Phillips et al. 2009; Austin and Van Niel 2011; Domisch et al. 2015; Wan et al. 2019; Čengić et al. 2020). The last one is particularly important because species are influenced by processes acting at different spatial resolutions and extents (i.e., the size of the area) (Wiens 1989; Pearson and Dawson 2003; Lowe et al. 2006). For example, climate is an important driver of species distribution at large extents and coarse resolutions, while variables like land use are expected to be more important at higher resolutions and smaller extents (Pearson and Dawson 2003). This implies that the choice of spatial resolution affects the relative importance of different predictors as well as the overall predictive performance of SDMs (Seo et al. 2009; Farashi and Alizadeh‐Noughani 2018; Kärcher et al. 2019; Wan et al. 2019). However, the evidence is not conclusive as some studies uncovered strong fluctuations in the predictive performance of SDMs across resolutions (Wan et al. 2019), while others reported limited effects (Farashi and Alizadeh‐Noughani 2018; Kärcher et al. 2019).

Further, research efforts on the effect of spatial resolution focused mainly on terrestrial ecosystems, leaving freshwater fish species understudied (but see Kärcher et al. 2019). Findings obtained for species in terrestrial ecosystems do not necessarily apply to species in freshwater habitats, since SDMs for terrestrial species are commonly based on grid cells, while SDMs of freshwater fish need to account the hierarchical structure of nested catchments in freshwater systems (Bean et al. 2014; Farashi and Alizadeh‐Noughani 2018; Seo et al. 2009). Importantly, freshwater conservation and assessment frameworks rely on hydrology‐based spatial units to delineate species ranges, identify priority areas and guide management actions, reinforcing the relevance of evaluating SDMs for freshwater fishes based on hydrologically meaningful spatial units and resolutions (Tedesco et al. 2017; IUCN SSC Red List Technical Working Group 2024; Côte et al. 2025; Ridley et al. 2025). Additionally, previous studies focused on the spatial resolution of freshwater fish SDMs covered only relatively small regions and few species (Kärcher et al. 2019; Schmidt et al. 2020). Hence the effects of spatial resolution on freshwater fish SDMs across large extents remains understudied. Because freshwater ecosystems worldwide suffer disproportionally from various anthropogenic pressures (Vörösmarty et al. 2010; Grill et al. 2019; Reid et al. 2019; Tickner et al. 2020; Barbarossa et al. 2021), there is urgency to establish large‐scale conservation measures to protect freshwaters and a growing interest in integrating SDM outputs into assessment frameworks such as from the IUCN Red List of Threatened Species (Santini et al. 2019; IUCN SSC Red List Technical Working Group 2024). As SDMs play an important role in conservation planning and biodiversity assessments for policy support (Peterson 2006; Catullo et al. 2008; Markovic et al. 2014; Warren et al. 2018; Araújo et al. 2019; Tao et al. 2023), it is key to understand how the choice of spatial resolution affects freshwater fish SDMs at large spatial extents, especially if they are to be used to estimate species ranges and support extinction risk assessments.

Here we aimed to analyse how the choice of spatial resolution affects the predictive performance, predictor variable importance and range size predictions of freshwater fish SDMs at large spatial extents. We considered five spatial resolution levels from the HydroSHEDS database (Lehner and Grill 2013), which correspond to the spatial units currently used in IUCN Red List of Threatened Species assessments of freshwater species (IUCN SSC Red List Technical Working Group 2024). At each resolution, we established the SDMs for 49 fish species across Europe based on occurrence data from the Global Biodiversity Information Facility (GBIF), a set of predictors representative of climate, hydrology and anthropogenic pressures and nine algorithms. We used GBIF because it is the largest repository of species occurrence data originating from many sources (Edwards et al. 2000; Yesson et al. 2007; Samy et al. 2013; Anderson et al. 2016). In line with the results of studies on terrestrial systems, we expected model performance to differ across resolutions (Seo et al. 2009; Farashi and Alizadeh‐Noughani 2018). In particular, as predictive performances may increase with the amount of data used to train the models (Gaul et al. 2020), we expected finer resolutions to result in better SDMs performances, as smaller spatial units are related to larger data quantities. Further, at coarser resolutions we expected an increase in importance of climatic variables (e.g., temperature) compared to other variables (e.g., land use), which we expected to have greater importance at finer resolutions (Pearson and Dawson 2003).

## 2 Methods

### 2.1 Study Area Extent and Spatial Resolution

For reasons of data availability, we focused on Europe (Figure S1). We used the global HydroSHEDS database (Lehner and Grill 2013) to identify hydrobasins and their sub‐catchments as spatial modelling units (Domisch et al. 2015). Hydrobasins represent hydrological catchments in which surface water drains to a common river. The hydrobasins follow a hierarchically nested structure, in which basins are subdivided into progressively smaller hydrological units at higher resolutions, reflecting the upstream–downstream connectivity of river networks where larger basins consist of multiple sub‐basins ultimately draining into the same river (Lehner and Grill 2013). We considered resolution levels 8 to 12, aligned with the levels used in IUCN range maps (IUCN SSC Red List Technical Working Group 2024). Level 8 has the coarsest resolution, containing 25,315 hydrobasin units within our study area extent with a median size of 485 km^2^, followed by 66,964 level 9 units (median size = 203 km^2^), 124,502 level 10 units (138 km^2^), 137,347 level 11 units (135 km^2^) and 137,658 level 12 units (135 km^2^) (Table S1).

### 2.2 Species Occurrence Data Extraction and Cleaning

We retrieved freshwater fish species occurrence data from the Global Biodiversity Information Facility (GBIF; https://www.gbif.org), extracting data for fish classes and orders reported in Fishbase (https://fishbase.se/tools/Classification/ClassificationList.php). We harmonised fish species names based on Fishbase (Froese and Pauly 2025) to avoid name mismatches and to resolve synonyms. We selected records within European HydroSHEDS basins only and removed observations without, or with likely erroneous, geographic coordinates, as indicated in GBIF data labels (Table S2). To avoid pseudoreplication, we considered one occurrence observation per species per sub‐basin for each of the spatial resolutions investigated (hydrobasins at level 8, 9, 10, 11 and 12). We excluded marine fish species from our analysis, based on species ecology information reported in Fishbase. Next, we filtered fish occurrence data for the period 1992–2015 to match the time range covered by the environmental predictors (see Section 2.3). Lastly, we selected only the species with at least 200 occurrence points at level 8 hydrobasins, resulting in a total of 49 species considered for this study (Table S3).

### 2.3 Environmental Data Selection and Processing

We selected environmental and anthropogenic predictors considered important for freshwater fish species distributions (Table S4). As environmental predictors, we included temperature, streamflow and topographic variables, which are key factors determining fish species distributions (Bond et al. 2015; Ruhí et al. 2015; Comte and Olden 2017; Carvajal‐Quintero et al. 2019; Barbarossa et al. 2021; Waldman and Quinn 2022). We used air temperature as a proxy of water temperature, in line with previous studies (Buisson et al. 2008; Lassalle et al. 2010; Wenger et al. 2011). We obtained annual mean temperature (BIO1), maximum temperature of the warmest month (BIO5), temperature annual range (BIO7) and mean temperature of the warmest quarter (BIO10) from WorldClim dataset (Fick and Hijmans 2017), to cover typical temperatures as well as temperature variability and extremes (Markovic et al. 2012, 2019; Kärcher et al. 2019). Similarly, we used mean, minimum and maximum yearly streamflow rates obtained from the FLO1K dataset (Barbarossa et al. 2018), as freshwater fish populations and biodiversity are influenced by the overall magnitude of flow as well as its extremes (Wenger et al. 2011; Bond et al. 2015; Ruhí et al. 2015; Schipper and Barbarossa 2022). For topography, we selected roughness, a measure of difference in terrain height that influences freshwater systems structure, groundwater flow and species dispersal (Pearson and Dawson 2003; Vidon and Hill 2004; Castelltort et al. 2009; Czarnota et al. 2014; Amatulli et al. 2018). We obtained terrain roughness from EarthEnv (Amatulli et al. 2018).

As anthropogenic variables, we included land use, human population density and the presence of dams. Land use and human population density can influence fish distributions through, for example, shoreline erosion, channel modifications (Urban and Rhoads 2003; Gregory 2011), or runoff of nutrients and other substances causing eutrophication and pollution (Dudgeon et al. 2006; Weijters et al. 2009; Utz et al. 2010; Waldman and Quinn 2022). Dams act as barriers, fragmenting freshwater fish habitats, isolating populations and hindering their migrations (Dudgeon et al. 2006; Van Puijenbroek et al. 2019; Barbarossa et al. 2020; Waldman and Quinn 2022; Keijzer et al. 2024). We extracted dam locations from the Global Dam Tracker (GDAT; Zhang and Gu 2023), yearly land use data from Climate Data Store (Copernicus Climate Change Service 2019) and yearly human population density from GlobPOP (Liu et al. 2024).

The input datasets for climate, streamflow and land use are year‐specific, so we calculated long‐term mean values over the period 1992–2015, representing the common time frame across all three variables. For dams, we retained those constructed before 2016. Next, we aggregated all predictor variables to each of the resolution levels 8, 9, 10, 11 and 12 of the hydrobasin map, resulting in five sets of predictor data representing five spatial resolutions. For cropland and built‐up area, we calculated the fractions of these land use types per hydrobasin unit. For temperature, streamflow, topography and human population density, we calculated the mean values per hydrobasin unit. Because upstream pressures may affect water quality downstream (Dudgeon et al. 2006; Chakraborti 2021), we also considered the mean human population density and area fractions of cropland and built‐up area in adjacent upstream basins as additional predictors. We calculated the density of dams per hydrobasin, as well as the density of dams of their adjacent upstream and downstream basins, because dams may act as barriers to both up and downstream fish migrations (Malmqvist and Rundle 2002; Carvajal‐Quintero et al. 2019; Van Puijenbroek et al. 2019; Barbarossa et al. 2020).

To reduce the risk of unstable or unreliable model relationships, we tested for collinearity among predictors, excluding one of each pair of variables with an absolute pairwise correlation coefficient (Pearson) of 0.7 or higher (Dormann et al. 2013). We found that annual mean temperature (BIO1), maximum temperature of the warmest month (BIO5) and mean temperature of the warmest quarter (BIO10) were collinear with each other, independently of the hydrobasin level. Additionally, mean streamflow was collinear with minimum streamflow and maximum streamflow and built‐up area within the catchment and upstream built‐up area were collinear with human population density within the catchment and upstream human population density, respectively. We kept annual mean temperature (BIO1), in combination with temperature annual range (BIO7), to account for both the average temperature conditions and the annual variability. Among the streamflow variables, we kept minimal streamflow, as previous studies indicated that low flow conditions impact fish populations stability (Bond et al. 2015; Ruhí et al. 2015). Lastly, we decided to keep built‐up area and remove human population density (both upstream and within‐catchment), as land use is directly connected to channel modification, changes in hydrological regimes and water quality (Gregory 2011; Wilkinson et al. 2018). Furthermore, land use data is obtained from satellite observations, while human population density is modelled and potentially subject to larger uncertainty (Copernicus Climate Change Service 2019; Liu et al. 2024).

### 2.4 SDM Fitting and Evaluation

**FIGURE 1:**
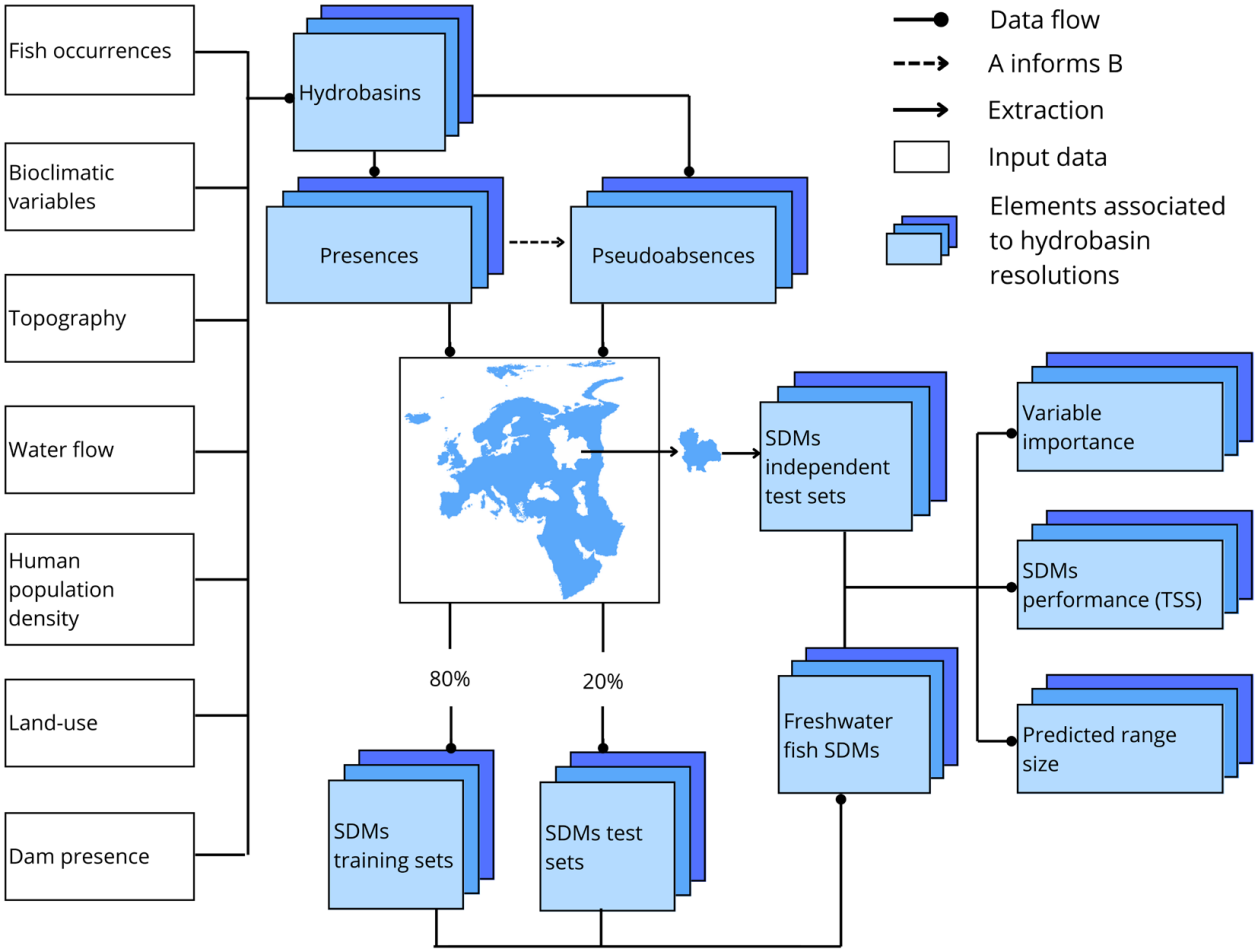
Schematic representation of the study's workflow.

For each of the five resolution levels studied and for each species, we selected pseudoabsence points from the hydrobasin units (i) without occurrence records of the focal species and (ii) included in level 4 basins where the species of focus occurs (i.e., level 4 basins containing at least one occurrence record). In total, we considered 240 level 4 basins, with a median size of 50,364 km^2^ (Table S1). To account for spatial bias in the GBIF presence records, we obtained pseudoabsences according to the target group approach by only sampling pseudoabsence points from hydrobasin units (resolutions 8 to 12) containing occurrence records of other species, but not the species of focus (Phillips et al. 2009; Barber et al. 2022). To test the predictive performance of our models, we split the data into two subsets: a subset to train the models and a geographically independent test subset (Figure 1). To obtain the independent test subset, we set aside data from a group of level 4 basins. To retain sufficient data for both the subsets, we selected a testing subset of level 4 basins which contained 10%–30% of presences and pseudoabsences for each species. We used the data from the remaining level 4 basins to train the models, limiting the number of pseudoabsences per species to 10,000 (Barbet‐Massin et al. 2012) and using 80% of the training data for model fitting and the remaining 20% for testing in a five‐fold cross‐validation (Figure 1).

We equally weighted presences and pseudoabsences in model fitting and employed nine algorithms commonly used in species distribution modelling (Seo et al. 2009; Farashi and Alizadeh‐Noughani 2018; Čengić et al. 2020). These included two classification methods (Classification Tree Analysis, CTA; Flexible Discriminate Analysis, FDA), three regression techniques (Generalised Linear Model, GLM; Generalised Additive Model, GAM; Multivariate Adaptive Regression Splines, MARS) and four machine‐learning techniques (Artificial Neural Network, ANN; Boosted Regression Trees, BRT; Random Forest, RF; Maximum Entropy, MaxEnt). Thus, we fitted nine models per species (*n* = 49) and spatial resolution (*n* = 5) and then created one ensemble model per species and spatial resolution combining all nine algorithms by weighting them based on their five‐fold cross‐validated True Skill Statistic (TSS). The TSS ranges from −1 to +1 (perfect prediction), where a value of 0 indicate performance similar to a modelled distribution based on random assignment of presence and absence (Allouche et al. 2006). We used TSS because it is widely used to evaluate SDM performance and it penalises both false positives and false negatives (Allouche et al. 2006). We also used the TSS to evaluate the predictive performance of the ensemble models based on the geographically independent test subset. To test for potential differences in the variance and mean of the TSS values across resolutions, we performed a Levene's test for homogeneity of variance, followed by an ANOVA.

To compare predictor variable importance among the different spatial resolutions, we used the ensemble models to make predictions based on permuted predictor variables, one variable at a time, and calculated the Pearson correlation between the predictions of the original model and the predictions of the model with the permuted variable (Smith and Santos 2020). We then refitted the models based on all training data and used these models to predict the species' distributions by binarising Probability of Occurrence (PoO) maps based on the PoO threshold that maximises TSS. We constrained the predicted distributions to level 4 hydrobasins with at least one occurrence of the focal species. Finally, we calculated the proportional difference in species range size between our predicted ranges and ranges derived directly from occurrence data. To obtain the latter, we summed the area size of the basins containing occurrence data.

For a systematic overview of the methodological aspects of the fitted SDMs, following the ODMAP protocol (Zurell et al. 2020), we refer to Table S5. We used R v4.3.2 and RStudio for data processing, model building and evaluation (R Core Team 2023; RStudio Team 2023). We used the packages ‘data.table’ (Barrett et al. 2006), ‘terra’ (Hijmans 2020) and ‘sf’ (Pebesma 2016) for data manipulation and analysis, ‘dismo’ (Hijmans et al. 2010) for the creation of bioclimatic variables and ‘biomod2’ (Thuiller et al. 2012) for establishing the SDMs.

## 3 Results

### 3.1 Model Predictive Performance

**FIGURE 2:**
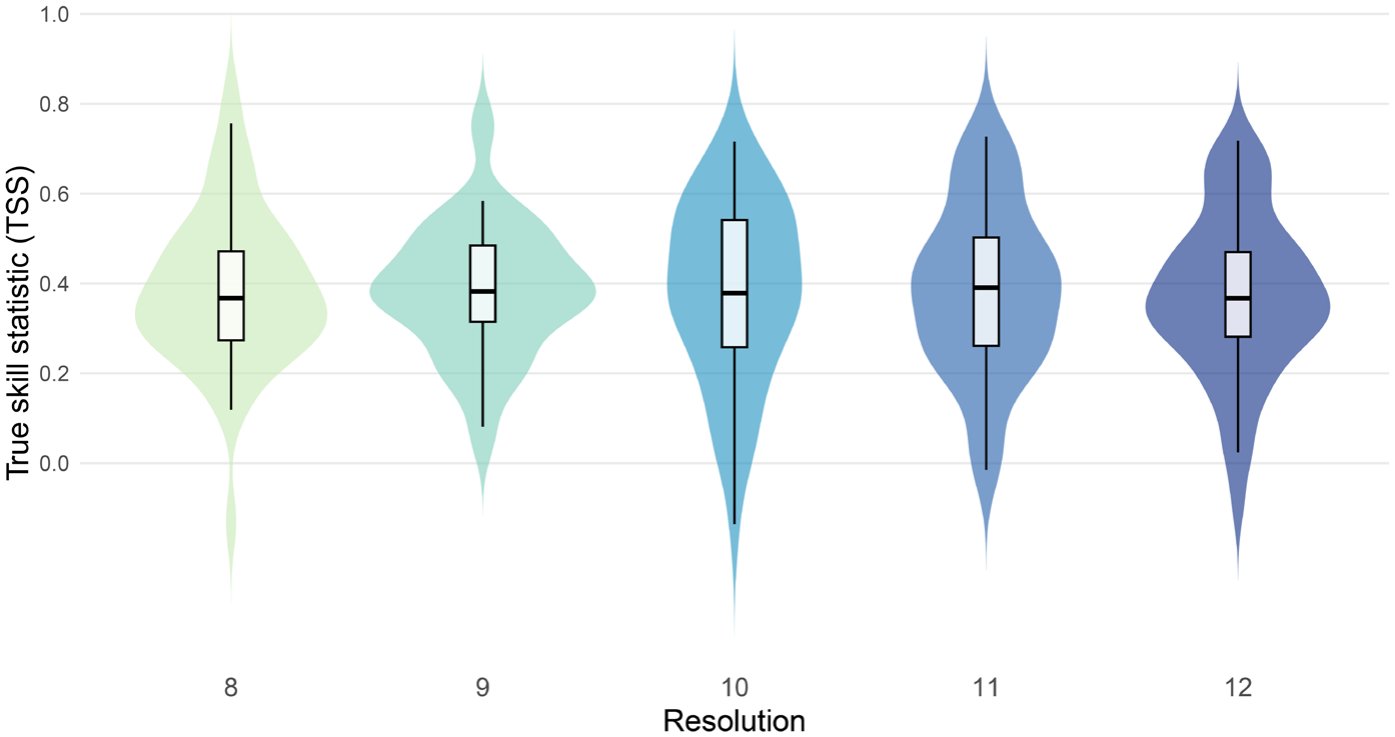
Distribution of the true skill statistic (TSS) values of ensemble species distribution models (SDMs) obtained in spatially independent validation across 49 freshwater fish species for each spatial resolution.

The predictive performance of the SDMs was highly similar across the resolutions, as revealed by the lack of statistically significant differences in the variance and mean of the TSS values (Levene's test *p*‐value = 0.22; repeated measures ANOVA *p*‐value = 0.98) (Figure 2). Median TSS values ranged from 0.37 to 0.39 across the different resolutions. SDMs built at resolution level 8 had a median TSS of 0.39 (95% range = 0.02–0.70) across the 49 species, followed by SDMs at level 9 with a median TSS of 0.38 (0.09–0.71), level 10 with 0.38 (0.00–0.69), level 11 with 0.39 (0.02–0.70) and level 12 with 0.37 (0.03–0.68). Models based on single algorithms reflected a similar pattern, generally exhibiting equivalent performances across resolutions (Figure S2). Among the algorithms, GBM showed overall the best performance (median TSS across all resolutions = 0.38; 95% range across all resolutions = 0.03–0.70), followed by MARS (0.36; −0.02 to 0.70), RF (0.35; −0.02 to 0.63), MAXENT (0.35; −0.01 to 0.67) and FDA (0.35; 0.01–0.70), CTA (0.34; 0.04–0.65) and GLM (0.34; −0.02 to 0.73), ANN (0.33; −0.05 to 0.68) and finally GAM (0.30; −0.07 to 0.70) (Table S6). At the species level, ensemble models for *Squalius pyrenaicus* showed the highest performance across resolutions, combined with a low variability (median TSS = 0.72; sd TSS = 0.04), while models for *Cottus poecilopus* showed one of the lowest performances with the highest variability (median TSS = 0.14; sd TSS = 0.26) (Table S7). Furthermore, models revealed increasing performance towards higher resolutions for some species, like *Abramis brama* (TSS level 8 = 0.34; TSS level 12 = 0.45) and opposite trends for others, like *Cyprinus carpio* (TSS level 8 = 0.47; TSS level 12 = 0.32).

### 3.2 Variable Importance

**FIGURE 3:**
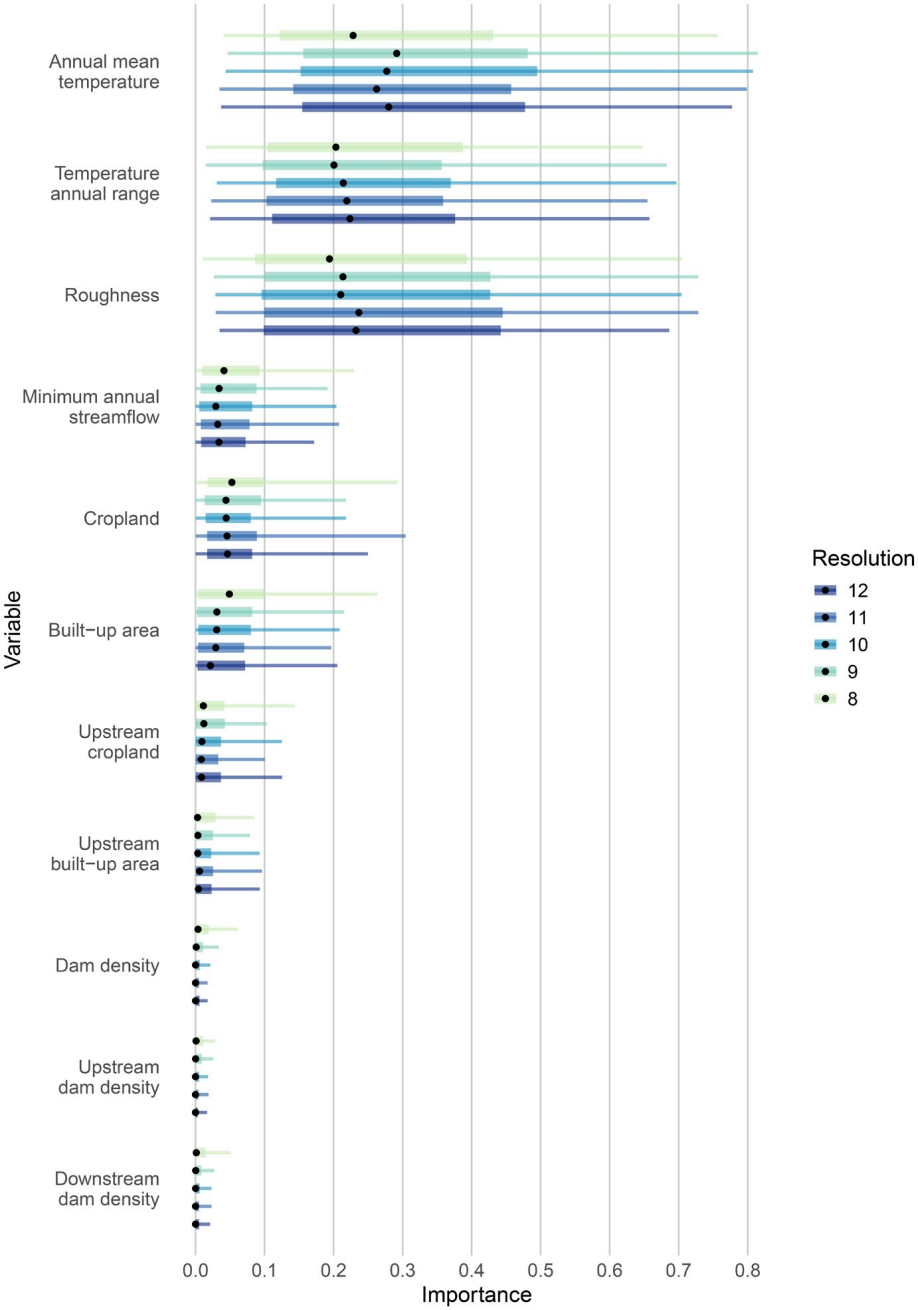
Variable importance values of ensemble species distribution models (SDMs) across 49 freshwater fish species for each spatial resolution. Black dots = median; boxes = IQR; whiskers = 95% range.

Similar to the TSS, the variable importance values showed little difference across spatial resolutions (Figure 3). Annual mean temperature (BIO1), temperature annual range (BIO7) and roughness showed the highest median importance values, followed by land use within the catchment and streamflow (Table S8). Dam density variables were the least important. Temperature and roughness variables showed the highest variability in importance across the species, highlighting they were important predictors for some species, yet poorly informative for others. This finding was again largely consistent across resolutions (Figure S3). BIO1 was the most important variable for 37%–43% of the species across resolutions. Following, roughness was the most important for 31%–37% of the species and BIO7 for 24%–27% of the species across resolutions. Streamflow and built‐up area within the catchment showed lower variability in their importance and were the most important variables only for a small fraction of the species at some resolution levels. Cropland within catchment, upstream land use and dam density were never found to be the most important variables.

### 3.3 Range Size

**FIGURE 4:**
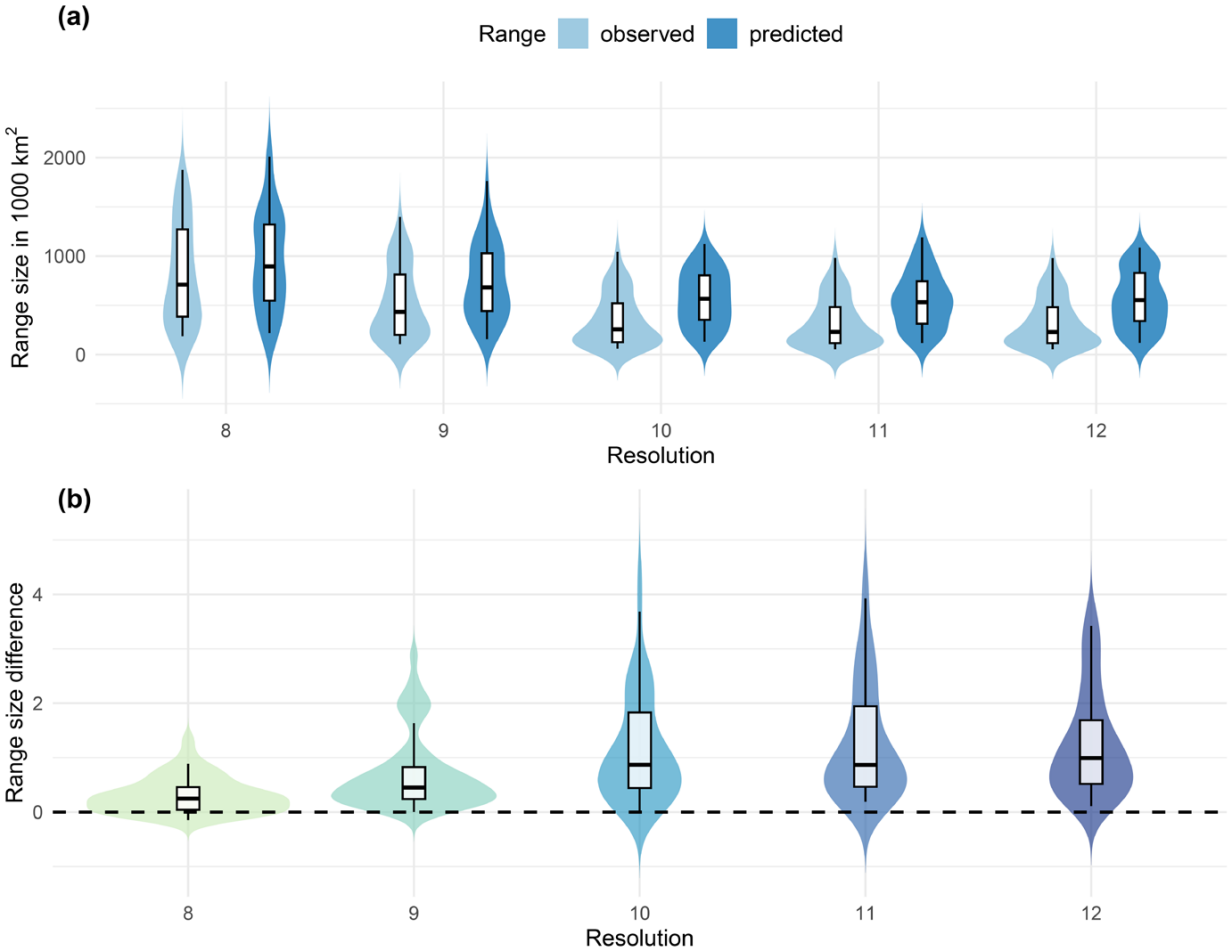
Observed and predicted range size from spatial predictions of ensemble species distribution models (SDMs) across 49 freshwater fish species for each spatial resolution (a) and relative difference between predicted and observed range size of freshwater fish species for each spatial resolution, calculated as (predicted range size – observed range size)/observed range size (b).

Both predicted and observed range size decreased towards higher resolutions (Figure 4a). Predicted median range sizes equalled 894,792 km^2^ (95% range = 236,874–1,803,966) at resolution level 8, 681,806 km^2^ (196,880–1,353,956) at level 9, 566,527 km^2^ (139,572–1,002,005) at level 10, 531,548 km^2^ (147,109–1,049,659) at level 11 and 551,992 km^2^ (144,568–988,197) at level 12. Observed range sizes were smaller compared to their predicted counterparts, with a median range size of 709,856 km^2^ (95% range = 207,135–1,659,187) at resolution level 8, 432,259 km^2^ (120,093–1,103,712) at level 9, 256,020 km^2^ (72,166–804,219) at level 10, 229,559 km^2^ (64,093–766,103) at level 11 and 228,384 km^2^ (63,467–765,325) at level 12. The relative difference between predicted and observed range size increased towards higher resolutions, from a median of 0.25 (95% range = −0.1 to 0.88) at level 8, to 0.45 (0.09–2.1) at level 9, 0.87 (0.08–2.66) at level 10 and at level 11 (0.21–3.21) and 0.99 (0.18–3.23) at level 12 (Figure 4b).

## 4 Discussion

### 4.1 Interpretation

In this study, we developed weighted ensemble SDMs for 49 freshwater fish species in Europe to examine the impact of the choice of spatial resolution on model predictive performance, variable importance and predicted range size. While we expected better predictive performance at higher resolutions (corresponding with a larger number of occurrence records), our analysis revealed no significant differences in predictive performance across the resolutions we analysed. Further, while we expected the importance of climatic variables to decrease and variables exhibiting more fine‐grain heterogeneity to increase at higher resolutions, the importance of all variables remained largely constant across resolutions. Among the variables we analysed, topography and temperature were the most important in predicting species distributions across all resolutions, while upstream land use and dam density variables were the least important. Topography and temperature showed, however, the largest variability in importance across species. Finally, we found that both predicted and observed species ranges decreased in size towards higher resolutions, while the difference between predicted and observed range sizes increased. Predicted ranges were consistently larger than observed ranges, with differences ranging from 25% at level 8 to 99% at level 12.

The negligible differences in predictive performance and variable importance across resolutions we observed in our analysis contrasted with previous studies on the effect of spatial resolution on SDMs, including both terrestrial and aquatic species (Graf et al. 2005; Kärcher et al. 2019; Markovic et al. 2019). The little differences we observed may indicate that the differences in hydrobasin sizes across the HydroSHEDS levels are too small to cause significant differences in predictive performance and variable importance, especially between levels 11 and 12 (Table S1). Averaging some of the environmental variables from the grid‐level to the hydrobasin‐level could also have decreased the possibility of finding differences in model performance and variable importance across resolutions. Taking the average likely reduces variable heterogeneity and variance within the hydrobasin units, which may impair the ability of the SDMs to discriminate suitable and unsuitable conditions within the hydrobasins. However, we found that the variability of the environmental variables is much larger between hydrobasins compared to the variability within hydrobasins for all environmental variables (except for streamflow) and at each hydrobasin level (see Supporting Information; Table S9). This suggests that ignoring variable heterogeneity within the hydrobasins units is ulikely to have had a large impact on our SDMs.

Still, even the hydrobasin units defined at level 12 may have been too coarse to reveal species distribution responses to local factors, like land use, which may be particularly impactful at finer resolutions. Therefore, the low importance of these variables does not imply ecological irrelevance. For example, the low importance of upstream land use in our models may reflect that nutrients and pollutants have the greatest impact near the emission source and may be partially absorbed, retained, or diluted before reaching downstream basins (Tian et al. 2011; Singh and Gupta 2016; Šaulys et al. 2019). This may also explain why we found that land use within catchment held greater importance than upstream land use, similar to what has been observed previously (Markovic et al. 2012). We note that previous studies on SDMs performance and variable importance in relation to spatial resolution returned contrasting results (Farashi and Alizadeh‐Noughani 2018; Kärcher et al. 2019; Wan et al. 2019). In combination with our findings, this indicates that follow‐up research is needed to better understand the influence of resolution on SDMs in relation to potentially relevant factors including unit size, spatial extent and species traits. Indeed, while predictive performance was stable when aggregated across species, we found different species‐specific changes in model performance in response to changes in resolution. Models for some species showed little differences between resolutions, while we found more variability for others, with increasing or declining performance towards higher resolutions. Thus, our findings are contingent on the sample of 49 species used in the modelling. Further by considering only species with at least 200 occurrence records at level 8 hydrobasins, species with very small ranges remained underrepresented and their response to spatial resolution poorly addressed in our analysis. Previous studies detected a few cases where variable importance differed more between species than between resolutions (Kärcher et al. 2019) and the large inter‐species variability we observed for the importance of topography and temperature underlines the relevance of follow‐up research into species traits and understudied species.

The low importance of dams in our SDMs is in contrast with previous studies which showed that dams severely fragment freshwater systems, limit species dispersion and may prompt fish biodiversity decline (Barbarossa et al. 2020; Merg et al. 2020; Liu et al. 2022; Keijzer et al. 2024). As only 27% of the species we analysed are diadromous, the limited importance of dams may reflect that the majority of species in our dataset are freshwater‐only species. Nevertheless, freshwater‐only species are also expected to be affected by habitat fragmentation due to dams (Barbarossa et al. 2020; Keijzer et al. 2024). We note that we did not consider the presence of freshwater system barriers not covered by dam datasets (Zhang and Gu 2023), such as small dams, weirs, culverts and bed‐sills which are more abundant than large‐size dams, but can still affect species movements and distributions (Jones et al. 2019, 2021; Belletti et al. 2020). Finally, both the hydrobasin units analysed and the point occurrence data might be too coarse to capture impacts of dams on broad‐scale fish distributions.

The changes in species range size across resolution are in line with former investigations on bird species that highlighted a decrease in predicted range size when moving to higher resolutions (Hurlbert and Jetz 2007). This trend may indicate that higher‐resolution models are better able to capture fine‐grain differences in species occurrence. At the same time, however, the increasing mismatch between predicted and observed range sizes towards higher resolutions points at an increasing probability of false positives. This, in turn, could reflect a genuine overestimation of species distribution as well as gaps in the occurrence data (false absences). In fact, despite being the largest occurrence dataset worldwide, GBIF remains incomplete (Edwards et al. 2000; Yesson et al. 2007; Samy et al. 2013; Anderson et al. 2016). In this context, we stress that we mapped the observed species range based on hydrobasins in which we found at least one observation record. False absences (i.e., a lack of observations for basins in which a species may occur) result in smaller observed species ranges, which is more likely to happen at finer resolutions. Therefore, incompleteness of observation records may explain that SDM ranges are larger in size than observed ranges, especially at finer resolutions. Additionally, the binarisation of the SDM predictions based on TSS may favour overpredictions if the number of species records is low (Leroy et al. 2018; Hellegers et al. 2025), which may also contribute to the predicted ranges being mostly larger than the observed ranges. Alternatively, the mismatch in range size could result from relatively large areas where important environmental factors, such as temperature and topography, show similar values, but only some basins host fish occurrences while others do not, possibly because of variables we did not consider in our analysis. The existence of unrecorded barriers may also contribute to the mismatch. Indeed, models may predict species occurrences in basins which are climatically suitable but out of reach because of these barriers.

### 4.2 Implications and Outlook

The small differences in predictive performance and variable importance across spatial resolutions observed in this study suggest that freshwater fish SDM performance and predictor importance are relatively robust to differences in spatial resolution. However, we detected a clear effect of the choice of resolution on modelled range size, with smaller predicted species ranges at higher resolutions. As previous investigations already suggested, this may indicate that higher resolutions provide a more accurate representation of the species range, avoiding areas where the species does not occur (Hurlbert and Jetz 2007) and that would otherwise be included in coarser resolution units. Thus, the larger predicted and observed range sizes at coarser resolutions might lead to an underestimation of extinction risk compared to higher resolutions, which might give a more accurate range estimate. Hence, while coarse‐grain SDMs might be useful for broad‐scale prioritisation of conservation or monitoring efforts, higher‐resolution SDMs might be more appropriate to identify species at risk of extinction, reasoning from a precautionary principle. Additionally, higher‐resolution SDMs might be more appropriate for small‐ranged and rare species, thanks to the larger number of spatial modelling units. However, comprehensive, detailed and precise data is needed to build high‐resolution SDMs, especially if these models are meant to be employed for conservation support.

Given that temperature and topography were important predictors in the SDMs across all the resolutions we analysed and that previous studies highlighted their influence on freshwater fish distribution and freshwater systems overall (Daufresne et al. 2004; Czarnota et al. 2014; Isaak et al. 2017; Carvajal‐Quintero et al. 2019), we recommend including variables related to these factors by default. In addition, given the high inter‐specific variability in variable importance, we also recommend tailoring the predictor variable selection to the species of focus. Finally, we stress the need to obtain and refine high‐quality data on other predictors that may be important for freshwater fish species, like the presence of dams and water quality parameters, as well as species occurrences through long‐lasting monitoring projects. Tailoring citizen science programs to cover data‐deficient areas and species and further synergising these initiatives with technologies like remote sensing, may enhance the quality of the data we need to develop adequate SDMs (Chandler et al. 2017; Feldman et al. 2021).

## Author Contributions

**Davide Fundaro':** conceptualization (supporting), data curation (lead), formal analysis (lead), investigation (lead), methodology (supporting), visualization (lead), writing – original draft (lead). **Jelle P. Hilbers:** conceptualization (equal), methodology (equal), supervision (equal), visualization (supporting), writing – review and editing (equal). **Koen J. J. Kuipers:** conceptualization (equal), methodology (equal), supervision (equal), visualization (supporting), writing – review and editing (equal). **Aafke M. Schipper:** conceptualization (equal), methodology (equal), project administration (lead), supervision (lead), visualization (supporting), writing – review and editing (equal).

## Funding

This work was supported by the Dutch Research Council (NWO, Grant VI.Vidi.213.093).

## Conflicts of Interest

The authors declare no conflicts of interest.

## Supporting information


**Data S1:** ece373472‐sup‐0001‐Supinfo.docx.
**Table S1:** Mean, median, interquartile range (IQR), 95% range (95%) size (in km^2^) and number of hydrobasin units per resolution level.
**Table S2:** Tags identifying GBIF data with potentially erratic coordinates. Tag description is provided at https://gbif.github.io/gbif‐api/apidocs/org/gbif/api/vocabulary/OccurrenceIssue.html.
**Table S3:** Freshwater only and diadromous fish species selected for the study and their extinction risk category according to the IUCN Red List of Threatened Species in parentheses.
**Table S4:** Environmental/anthropogenic variables selected for the study, their original spatial and temporal resolution, original time frame covered and data source.
**Table S5:** Overview of the SDMs fit in this study following the ODMAP protocol (Zurell et al. 2020).
**Table S6:** Distribution of model performance values across 49 freshwater fish species per algorithm and resolution level. Each cell contains the median true skill statistic (TSS) (top) and the 95% range (bottom).
**Table S7:** True skill statistic (TSS) values of the ensemble SDMs at each resolution level (8, 9, 10, 11, 12) and standard deviation (sd) across resolutions.
**Table S8:** Distribution of variable importance values from ensemble species distribution models (SDMs) across 49 freshwater fish species for each resolution level. Each cell contains the median importance (top), the interquartile range (centre) and the 95% range (bottom).
**Table S9:** Variables' inter‐basin squared sum (SS between), intra‐basin squared sum (SS within) and respective proportions over total variability across resolutions.
**Figure S1:** Study area (in blue).
**Figure S2:** Predictive performance as true skill statistic (TSS) of algorithms used to employ species distribution models (SDMs) for 49 freshwater fish species across resolutions 8 (a), 9 (b), 10 (c), 11 (d), 12 (e).
**Figure S3:** Proportion of freshwater fish species for which a given variable was the most important for each resolution level.

## Data Availability

The data sources and R code that support the findings of this study are available in Zenodo (https://doi.org/10.5281/zenodo.17376322).
