# Supplementary material for: How the Choice of Spatial Resolution Affects Freshwater Fish Species Distribution Models
Source: Ecol Evol. 2026 Apr 22;16(4):e73472. doi: 10.1002/ece3.73472 (PMC13100889; doi:10.1002/ece3.73472)
Supplement: Supplementary file 1 — Data S1: ece373472‐sup‐0001‐Supinfo.docx. Table S1: Mean, median, interquartile range (IQR), 95% range (95%) size (in km2) and number of hydrobasin units per resolution level. Table S2: Tags identifying GBIF data with potentially erratic coordinates. Tag description is provided at https://gbif.github.io/gbif‐api/apidocs/org/gbif/api/vocabulary/OccurrenceIssue.html. Table S3: Freshwater only and diadromous fish species selected for the study and their extinction risk category according to the IUCN Red List of Threatened Species in parentheses. Table S4: Environmental/anthropogenic variables selected for the study, their original spatial and temporal resolution, original time frame covered and data source. Table S5: Overview of the SDMs fit in this study following the ODMAP protocol (Zurell et al. 2020). Table S6: Distribution of model performance values across 49 freshwater fish species per algorithm and resolution level. Each cell contains the median true skill statistic (TSS) (top) and the 95% range (bottom). Table S7: True skill statistic (TSS) values of the ensemble SDMs at each resolution level (8, 9, 10, 11, 12) and standard deviation (sd) across resolutions. Table S8: Distribution of variable importance values from ensemble species distribution models (SDMs) across 49 freshwater fish species for each resolution level. Each cell contains the median importance (top), the interquartile range (centre) and the 95% range (bottom). Table S9: Variables' inter‐basin squared sum (SS between), intra‐basin squared sum (SS within) and respective proportions over total variability across resolutions. Figure S1: Study area (in blue). Figure S2: Predictive performance as true skill statistic (TSS) of algorithms used to employ species distribution models (SDMs) for 49 freshwater fish species across resolutions 8 (a), 9 (b), 10 (c), 11 (d), 12 (e). Figure S3: Proportion of freshwater fish species for which a given variable was the most important for each resolution level. [file ECE3-16-e73472-s001.docx]

**Supplementary Information**

**How the choice of spatial resolution affects freshwater fish species distribution models**

Supplementary tables

**Table S1**: Mean, median, interquartile range (IQR), 95% range (95%) size (in km^2^), and number of hydrobasin units per resolution level.

| **Resolution level** | **Size (km^2^)** | | | | **Number of units** |
| --- | --- | --- | --- | --- | --- |
|  | **Mean** | **Median** | **IQR** | **95% range** |  |
| 4 | 74,412.1 | 50,364.2 | 19,943.9 – 97,593.4 | 373.6 – 310,652.5 | 240 |
| 8 | 705.5 | 484.9 | 225 – 957.1 | 8.6 – 2515 | 25,315 |
| 9 | 266.7 | 203.1 | 113.3 – 368.6 | 4.5 – 789.4 | 66,964 |
| 10 | 143.4 | 138.4 | 103.6 – 177.1 | 4.7 – 351.4 | 124,502 |
| 11 | 130 | 135 | 101.6 – 166.6 | 4.3 – 239.6 | 137,347 |
| 12 | 129.7 | 134.9 | 101.6 – 166.4 | 4.3 – 238.8 | 137,658 |

**Table S2**: Tags identifying GBIF data with potentially erratic coordinates. Tag description is provided at https://gbif.github.io/gbif-api/apidocs/org/gbif/api/vocabulary/OccurrenceIssue.html.

| ZERO_COORDINATE |
| --- |
| PRESUMED_SWAPPED_COORDINATE |
| PRESUMED_NEGATED_LONGITUDE |
| PRESUMED_NEGATED_LATITUDE |
| GEODETIC_DATUM_INVALID |
| COORDINATE_REPROJECTION_SUSPICIOUS |
| COORDINATE_REPROJECTION_FAILED |
| COORDINATE_OUT_OF_RANGE |
| COORDINATE_INVALID |

**Table S3**: Freshwater only and diadromous fish species selected for the study, and their extinction risk category according to the IUCN Red List of Threatened Species in parentheses.

| **Migratory behaviour** | **Species list** |
| --- | --- |
| Freshwater only | *Abramis brama* (LC), *Achondrostoma arcasii* (NT), *Alburnoides bipunctatus* (LC), *Alburnus alburnus* (LC), *Ameiurus melas* (LC), *Barbatula barbatula* (LC), *Blicca bjoerkna* (LC), *Carassius auratus* (LC), *Chondrostoma nasus* (NT), *Cobitis paludica* (LC), *Cottus gobio* (LC), *Cottus poecilopus* (LC), *Ctenopharyngodon idella* (LC), *Cyprinus carpio* (LC), *Gambusia holbrooki* (LC), *Gobio lozanoi* (LC), *Gymnocephalus cernua* (LC), *Lepomis gibbosus* (LC), *Leucaspius delineates* (LC), *Leuciscus aspius* (LC), *Leuciscus idus* (LC), *Leuciscus leuciscus* (LC), *Leucos aula* (NT), *Lota lota* (LC), *Micropterus salmoides* (LC), *Misgurnus fossilis* (LC), *Phoxinus phoxinus* (LC), *Pseudorasbora parva* (LC), *Rhodeus amarus* (LC), *Sander lucioperca* (LC), *Squalius alburnoides* (LC), *Squalius cephalus* (LC), *Squalius pyrenaicus* (VU), *Telestes souffia* (LC), *Thymallus thymallus* (LC), *Tinca tinca* (LC) |
| Diadromous | *Chelon labrosus* (NT), *Coregonus Albula* (LC), *Coregonus lavaretus* (LC), *Coregonus maraena* (LC), *Gasterosteus aculeatus* (LC), *Oncorhynchus mykiss* (LC), *Osmerus eperlanus* (LC), *Petromyzon marinus* (LC), *Platichthys flesus* (LC), *Pungitius pungitius* (LC), *Salmo salar* (NT), *Salvelinus alpinus* (VU), *Salvelinus fontinalis* (LC) |

**Table S4**: Environmental/anthropogenic variables selected for the study, their original spatial and temporal resolution, original time frame covered, and data source.

| **Variable** | **Original spatial resolution** | **Original temporal resolution** | **Original time frame covered** | **Data source** |
| --- | --- | --- | --- | --- |
| Annual mean temperature (BIO1) | 2.5 minutes | 1 month | 1960 - 2021 | WorldClim |
| Mean temperature of the warmest quarter (BIO10) | 2.5 minutes | 1 month | 1960 - 2021 | WorldClim |
| Maximum temperature of the warmest month (BIO5) | 2.5 minutes | 1 month | 1960 - 2021 | WorldClim |
| Temperature Annual Range (BIO7) | 2.5 minutes | 1 month | 1960 - 2021 | WorldClim |
| Roughness | 1 km | NA | NA | EarthEnv |
| Mean annual streamflow | 1 km | 1 y | 1960 - 2015 | FLO1K |
| Minimum annual streamflow | 1 km | 1 y | 1960 - 2015 | FLO1K |
| Maximum annual streamflow | 1 km | 1 y | 1960 - 2015 | FLO1K |
| Local human population density | 1 km | 1 y | 1990 - 2020 | GlobPOP |
| Upstream human population density | 1 km | 1 y | 1990 - 2020 | GlobPOP |
| Local proportion of crops | 30 m | 1 y | 1992 - 2022 | Climate Data Store |
| Upstream proportion of crops | 30 m | 1 y | 1992 - 2022 | Climate Data Store |
| Local proportion of built up areas | 30 m | 1 y | 1992 - 2022 | Climate Data Store |
| Upstream proportion of built up areas | 30 m | 1 y | 1992 - 2022 | Climate Data Store |
| Local dam density | NA | NA | NA | Global Dam Tracker |
| Upstream dam density | NA | NA | NA | Global Dam Tracker |
| Downstream dam density | NA | NA | NA | Global Dam Tracker |

**Table S5**: Overview of the SDMs fit in this study following the ODMAP protocol (Zurell et al., 2020).

| **ODMAP element** | **Content** | **Where addressed** |
| --- | --- | --- |
| Overview | | |
| Study objective | Explore the effect of spatial resolutions, used in IUCN assessments, on SDM performance, variable importance, and predicted range size | Introduction; Methods 2.1 |
| Taxon | 49 freshwater fish species | Introduction; Methods; TableS3 |
| Location | Europe | Methods 2.1; Figure S1 |
| Spatial units | Hydrologically defined basins (HydroBASINS) | Methods 2.1 |
| Resolutions | HydroBASINS levels 8–12 | Methods 2.1; Table S1 |
| Temporal extent | 1992-2015 | Methods 2.2-2.3; TableS5 |
| Biodiversity data type | Point occurrence | Methods 2.2 |
| Type of predictors | climatic, topographic, hydrological, anthropogenic | Methods 2.3 |
| Conceptual model/hypotheses | Climate, topography, hydrology and anthropogenic pressures are correlated with freshwater fish species distributions | Methods 2.3 |
| Model algorithms | Cta, fda, glm, gam, mars, ann, brt, rf, maxent | Methods 2.4 |
| Use of ensemble modelling | We used ensemble models where the single algorithms were weighted based on their TSS during cross-validation | Methods 2.4 |
| Model workflow | We modelled freshwater fish species distributions across five spatial resolutions defined by hydrologically nested HydroBASINS levels (8–12). We aggregated species occurrence data to hydrobasin units, retaining one presence per species per basin at each resolution. We generated pseudoabsences using a target-group approach sampling within level 4 basins where the focal species occurred. For each species and spatial resolution, we spatially partitioned the data by withholding some level 4 basins as an independent test set, while we used the remaining data for model calibration with five-fold cross-validation. We fitted nine species distribution modelling algorithms using a common set of environmental and anthropogenic predictors aggregated to each resolution. We assessed predictor collinearity using pairwise Pearson correlations, excluding correlated variables (\|r\| ≥ 0.7) prior to model fitting. We evaluated models using the True Skill Statistic (TSS), and combined algorithm-specific predictions into ensemble models weighted by cross-validated TSS values. We evaluated final ensemble models performances against the independent test data, binarized using TSS-optimizing thresholds, and used them to estimate species range sizes and their relative differences across spatial resolutions. | Methods |
| Software | R v.4.3.2, biomod2, terra, sf, dismo, data.table | Methods 2.4 |
| Data | | |
| Taxonomic reference system | Fishbase | Methods 2.2 |
| Ecological level | Species | Methods 2.2 |
| Biodiversity data source | GBIF | Methods 2.2 |
| Sample size | ≥200 or higher occurrences per species at HydroBASINS level 8 | Methods 2.2 |
| Absence data | Pseudoabsences (target-group approach) obtained from HydroBASINS level 4 hosting at least one occurrence of the focal species | Methods 2.4 |
| Details on data cleaning | We filtered out occurrences without coordinates, or with likely erroneous coordinates | Methods 2.2; Table S3 |
| Potential errors and biases | Occurrence data partially obtained from easily accessible sites |  |
| Predictor variables | annual mean temperature (BIO1), maximum temperature of the warmest month (BIO5), temperature annual range (BIO7), mean temperature of the warmest quarter (BIO10), roughness, mean streamflow, minimum streamflow, maximum streamflow, human population density, upstream human population density, proportion of crops, upstream proportion of crops, proportion of built-up area, proportion of built-up area, dam density, upstream dam density, downstream dam density | Methods 2.3 |
| Predictor data source | WorldClim, FLO1K, EarthEnv, GlobPOP, Copernicus, GDAT | Methods 2.3 |
| Coordinate reference system | WGS 1984 |  |
| Predictor data processing | - We obtained bioclimatic variables (BIO1, BIO5, BIO7, BIO10) processing temperature and precipitation data through the package “dismo”. We obtained the average of each of the bioclimatic variables across the period 1992-2015 for each hydrobasin unit. - We obtained the average topography for each hydrobasin unit. - We obtained the average of mean, minimum, and maximum streamflow across the period 1992-2015 for each hydrobasin unit. - We obtained the average human population density across the period 1992-2015 for each hydrobasin unit. - We obtained the proportion of crops and built-up area from Copernicus and calculated their average across the period 1992-2015 for each hydrobasin unit. - We filtered out all the dams constructed since 2016 and calculated the density of dams per each hydrobasin unit. | Methods 2.3 |
| Model | | |
| Variable preselection | Predictors were preselected based on their importance according to previous studies on freshwater fish SDMs | Methods 2.3 |
| Multicollinearity | We assessed predictor collinearity using pairwise Pearson correlations, excluding correlated variables (\|r\| ≥ 0.7) | Methods 2.4 |
| Model settings | We fitted all the algorithms on training data in a 5-folds cross-validation where 20% of training data was randomly set aside for testing the algorithms performances. We refitted the ensemble model resulting from these algorithms on all the training data and permuted the variables to obtain their importance. We then refitted the ensemble model on all data and obtained the predictions for the study area constraining the predictions in level 4 basins hosting at least one occurrence of the focal species. | Methods 2.4 |
| Model averaging/ensembles | We weighted the algorithms according to their True Skill Statistic (TSS). | Methods 2.4 |
| Assessment | | |
| Performance statistics | We used the True Skill Statistic (TSS) to quantify model’s predictive performances. | Methods 2.4 |
| Prediction output | We used the ensemble models to predict the distribution range of freshwater fish species in the study area, and compared these predicted ranges with their observed counterparts. We predicted species distributions inside resolution level 4 hydrobasins hosting at least one occurrence of the focal species. | Methods 2.4 |

**Table S6**: Distribution of model performance values across 49 freshwater fish species per algorithm and resolution level. Each cell contains the median true skill statistic (TSS) (top) and the 95% range (bottom).

| **Algorithm** | **Resolution level 8** | **Resolution level 9** | **Resolution level 10** | **Resolution level 11** | **Revolution level 12** |
| --- | --- | --- | --- | --- | --- |
| ANN | 0.30  -0.17 – 0.67 | 0.34  0.02 – 0.63 | 0.40  -0.05 – 0.65 | 0.33  -0.04 – 0.69 | 0.34  0.07 – 0.64 |
| CTA | 0.35  0.08 – 0.63 | 0.35  0.13 – 0.61 | 0.34  0.03 – 0.61 | 0.35  0.04 – 0.68 | 0.34  0.08 – 0.66 |
| FDA | 0.34  -0.04 – 0.70 | 0.35  0.07 – 0.65 | 0.39  -0.03 – 0.63 | 0.34  0.03 – 0.70 | 0.33  0.04 – 0.68 |
| GAM | 0.26  -0.01 – 0.72 | 0.30  0.02 – 0.64 | 0.35  -0.16 – 0.61 | 0.29  -0.06 – 0.53 | 0.32  -0.05 – 0.67 |
| GBM | 0.38  0.06 – 0.74 | 0.38  0.11 – 0.68 | 0.39  0.02 – 0.67 | 0.39  0.04 – 0.68 | 0.40  0.03 – 0.66 |
| GLM | 0.32  0.03 – 0.69 | 0.32  0.09 – 0.70 | 0.35  -0.04 – 0.61 | 0.37  -0.03 – 0.73 | 0.34  0.00 – 0.73 |
| MARS | 0.33  -0.01 – 0.74 | 0.37  0.06 – 0.70 | 0.38  -0.04 – 0.63 | 0.36  0.01 – 0.70 | 0.35  0.00 – 0.67 |
| MAXENT | 0.32  -0.02 – 0.59 | 0.36  0.01 – 0.60 | 0.38  -0.01 – 0.62 | 0.35  0.04 – 0.69 | 0.35  0.01 – 0.64 |
| RF | 0.37  -0.07 – 0.62 | 0.35  0.03 – 0.59 | 0.33  0.02 – 0.60 | 0.35  0.10 – 0.69 | 0.34  0.01 – 0.63 |

**Table S7**: True skill statistic (TSS) values of the ensemble SDMs at each resolution level (8, 9, 10, 11, 12) and standard deviation (sd) across resolutions.

| **Species** | **8** | **9** | **10** | **11** | **12** | **sd** |
| --- | --- | --- | --- | --- | --- | --- |
| *Abramis brama* | 0.335 | 0.409 | 0.415 | 0.449 | 0.452 | 0.047 |
| *Achondrostoma arcasii* | -0.131 | 0.081 | 0.258 | 0.298 | 0.356 | 0.198 |
| *Alburnoides bipunctatus* | 0.463 | 0.234 | 0.314 | 0.206 | 0.282 | 0.100 |
| *Alburnus alburnus* | 0.274 | 0.351 | 0.316 | 0.399 | 0.415 | 0.058 |
| *Ameiurus melas* | 0.551 | 0.451 | 0.379 | 0.247 | 0.376 | 0.112 |
| *Barbatula barbatula* | 0.678 | 0.459 | 0.355 | 0.264 | 0.287 | 0.169 |
| *Blicca bjoerkna* | 0.419 | 0.444 | 0.570 | 0.463 | 0.602 | 0.081 |
| *Carassius auratus* | 0.529 | 0.367 | 0.208 | 0.232 | 0.267 | 0.131 |
| *Chelon labrosus* | 0.487 | 0.753 | 0.656 | 0.647 | 0.518 | 0.109 |
| *Chondrostoma nasus* | 0.270 | 0.399 | 0.280 | 0.402 | 0.480 | 0.089 |
| *Cobitis paludica* | 0.677 | 0.511 | 0.376 | 0.630 | 0.596 | 0.118 |
| *Coregonus albula* | 0.349 | 0.147 | 0.434 | 0.513 | 0.282 | 0.141 |
| *Coregonus lavaretus* | 0.627 | 0.274 | 0.165 | 0.673 | 0.210 | 0.241 |
| *Coregonus maraena* | 0.373 | 0.485 | 0.460 | 0.480 | 0.307 | 0.078 |
| *Cottus gobio* | 0.372 | 0.426 | 0.195 | 0.159 | 0.426 | 0.129 |
| *Cottus poecilopus* | 0.137 | 0.356 | -0.136 | 0.442 | -0.086 | 0.257 |
| *Ctenopharyngodon idella* | 0.231 | 0.382 | 0.339 | 0.251 | 0.413 | 0.080 |
| *Cyprinus carpio* | 0.469 | 0.404 | 0.351 | 0.345 | 0.324 | 0.058 |
| *Gambusia holbrooki* | 0.310 | 0.376 | 0.226 | 0.391 | 0.523 | 0.109 |
| *Gasterosteus aculeatus* | 0.472 | 0.442 | 0.619 | 0.705 | 0.661 | 0.117 |
| *Gobio lozanoi* | 0.339 | 0.217 | 0.065 | 0.346 | 0.261 | 0.114 |
| *Gymnocephalus cernua* | 0.324 | 0.523 | 0.355 | 0.344 | 0.466 | 0.087 |
| *Lepomis gibbosus* | 0.358 | 0.382 | 0.503 | 0.351 | 0.333 | 0.068 |
| *Leucaspius delineatus* | 0.394 | 0.493 | 0.378 | 0.385 | 0.383 | 0.049 |
| *Leuciscus aspius* | 0.455 | 0.392 | 0.692 | 0.533 | 0.467 | 0.115 |
| *Leuciscus idus* | 0.415 | 0.231 | 0.541 | 0.539 | 0.327 | 0.135 |
| *Leuciscus leuciscus* | 0.528 | 0.488 | 0.558 | 0.372 | 0.465 | 0.071 |
| *Leucos aula* | 0.293 | 0.189 | 0.047 | 0.019 | 0.170 | 0.112 |
| *Lota lota* | 0.318 | 0.547 | 0.603 | 0.585 | 0.094 | 0.220 |
| *Micropterus salmoides* | 0.265 | 0.363 | 0.535 | 0.261 | 0.667 | 0.178 |
| *Misgurnus fossilis* | 0.757 | 0.557 | 0.568 | 0.503 | 0.377 | 0.137 |
| *Oncorhynchus mykiss* | 0.356 | 0.410 | 0.142 | 0.241 | 0.334 | 0.106 |
| *Osmerus eperlanus* | 0.245 | 0.515 | 0.527 | 0.440 | 0.367 | 0.116 |
| *Petromyzon marinus* | 0.367 | 0.248 | 0.493 | 0.427 | 0.344 | 0.092 |
| *Phoxinus phoxinus* | 0.201 | 0.215 | -0.001 | 0.072 | 0.168 | 0.092 |
| *Platichthys flesus* | 0.462 | 0.344 | 0.684 | 0.436 | 0.598 | 0.136 |
| *Pseudorasbora parva* | 0.242 | 0.320 | 0.493 | 0.270 | 0.376 | 0.099 |
| *Pungitius pungitius* | 0.526 | 0.520 | 0.573 | 0.612 | 0.488 | 0.049 |
| *Rhodeus amarus* | 0.537 | 0.175 | 0.339 | 0.291 | 0.257 | 0.136 |
| *Salmo salar* | 0.272 | 0.315 | 0.110 | 0.191 | 0.152 | 0.084 |
| *Salvelinus alpinus* | 0.824 | 0.584 | 0.615 | 0.687 | 0.674 | 0.092 |
| *Salvelinus fontinalis* | 0.261 | 0.360 | 0.425 | 0.194 | 0.470 | 0.114 |
| *Sander lucioperca* | 0.294 | 0.300 | 0.445 | 0.263 | 0.303 | 0.071 |
| *Squalius alburnoides* | 0.119 | 0.041 | 0.018 | 0.021 | 0.033 | 0.042 |
| *Squalius cephalus* | 0.179 | 0.570 | 0.228 | 0.460 | 0.202 | 0.176 |
| *Squalius pyrenaicus* | 0.640 | 0.748 | 0.716 | 0.727 | 0.718 | 0.041 |
| *Telestes souffia* | 0.407 | 0.320 | 0.554 | 0.698 | 0.686 | 0.167 |
| *Thymallus thymallus* | 0.320 | 0.346 | 0.333 | -0.015 | 0.024 | 0.181 |
| *Tinca tinca* | 0.429 | 0.386 | 0.414 | 0.416 | 0.365 | 0.026 |

**Table S8**: Distribution of variable importance values from ensemble species distribution models (SDMs) across 49 freshwater fish species for each resolution level. Each cell contains the median importance (top), the interquartile range (centre), and the 95% range (bottom).

| **Variable** | **Resolution level 8** | **Resolution level 9** | **Resolution level 10** | **Resolution level 11** | **Resolution level 12** |
| --- | --- | --- | --- | --- | --- |
| Annual mean temperature (BIO1) | 0.23  0.12 – 0.43  0.04 – 0.76 | 0.29  0.16 – 0.48  0.05 – 0.81 | 0.28  0.15 – 0.50  0.04 – 0.81 | 0.26  0.14 – 0.46  0.03 – 0.80 | 0.28  0.15 – 0.48  0.04 – 0.78 |
| Temperature annual range (BIO7) | 0.20  0.10 – 0.39  0.02 – 0.65 | 0.20  0.10 – 0.36  0.02 – 0.68 | 0.21  0.12 – 0.37  0.03 – 0.70 | 0.22  0.10 – 0.36  0.02 – 0.65 | 0.22  0.11 – 0.38  0.02 – 0.66 |
| Roughness | 0.19  0.09 – 0.39  0.01 – 0.71 | 0.21  0.10 – 0.43  0.03 – 0.73 | 0.21  0.10 – 0.43  0.03 – 0.70 | 0.24  0.10 – 0.45  0.03 – 0.73 | 0.23  0.10 – 0.44  0.03 – 0.69 |
| Minimum annual streamflow | 0.04  0.01 – 0.09  0.00 – 0.23 | 0.03  0.01 – 0.09  0.00 – 0.19 | 0.03  0.01 – 0.08  0.00 – 0.20 | 0.03  0.01 – 0.08  0.00 – 0.21 | 0.03  0.01 – 0.07  0.00 – 0.17 |
| Local proportion of crops | 0.05  0.02 – 0.10  0.00 – 0.29 | 0.04  0.01 – 0.09  0.00 – 0.22 | 0.04  0.01 – 0.08  0.00 – 0.22 | 0.05  0.02 – 0.09  0.00 – 0.30 | 0.05  0.02 – 0.08  0.00 – 0.25 |
| Upstream proportion of crops | 0.01  0.00 – 0.04  0.00 – 0.14 | 0.01  0.00 – 0.04  0.00 – 0.10 | 0.01  0.00 – 0.04  0.00 – 0.13 | 0.01  0.00 – 0.03  0.00 – 0.10 | 0.01  0.00 – 0.04  0.00 – 0.13 |
| Local proportion of built up areas | 0.05  0.00 – 0.1  0.00 – 0.26 | 0.03  0.00 – 0.08  0.00 – 0.21 | 0.03  0.00 – 0.08  0.00 – 0.21 | 0.03  0.00 – 0.07  0.00 – 0.20 | 0.02  0.00 – 0.07  0.00 – 0.21 |
| Upstream proportion of built up areas | 0.00  0.00 – 0.03  0.00 – 0.09 | 0.00  0.00 – 0.03  0.00 – 0.08 | 0.00  0.00 – 0.02  0.00 – 0.09 | 0.01  0.00 – 0.03  0.00 – 0.10 | 0.00  0.00 – 0.02  0.00 – 0.09 |
| Local dam density | 0.00  0.00 – 0.02  0.00 – 0.06 | 0.00  0.00 – 0.01  0.00 – 0.03 | 0.00  0.00 – 0.01  0.00 – 0.02 | 0.00  0.00 – 0.00  0.00 – 0.02 | 0.00  0.00 – 0.01  0.00 – 0.02 |
| Upstream dam density | 0.00  0.00 – 0.01  0.00 – 0.03 | 0.00  0.00 – 0.01  0.00 – 0.03 | 0.00  0.00 – 0.01  0.00 – 0.02 | 0.00  0.00 – 0.00  0.00 – 0.02 | 0.00  0.00 – 0.00  0.00 – 0.02 |
| Downstream dam density | 0.00  0.00 – 0.01  0.00 – 0.05 | 0.00  0.00 – 0.01  0.00 – 0.03 | 0.00  0.00 – 0.01  0.00 – 0.02 | 0.00  0.00 – 0.00  0.00 – 0.02 | 0.00  0.00 – 0.01  0.00 – 0.02 |

**Table S9**: Variables’ inter-basin squared sum (SS between), intra-basin squared sum (SS within), and respective proportions over total variability across resolutions.

| **Variable** | **Resolution** | **SS between** | **SS within** | **SS between proportion** | **SS within proportion** |
| --- | --- | --- | --- | --- | --- |
| Annual mean temperature (BIO1) | 8 | 80,552,176 | 804,942.3 | 0.99 | 0.01 |
| Annual mean temperature (BIO1) | 9 | 80,807,212 | 549,905.8 | 0.99 | 0.01 |
| Annual mean temperature (BIO1) | 10 | 80,936,723 | 420,395.0 | 0.991 | 0.01 |
| Annual mean temperature (BIO1) | 11 | 80,963,100 | 394,018.2 | 0.995 | 0.005 |
| Annual mean temperature (BIO1) | 12 | 80,963,893 | 393,225.2 | 0.995 | 0.005 |
| Temperature annual range (BIO7) | 8 | 36,619,475 | 638,799.6 | 0.98 | 0.02 |
| Temperature annual range (BIO7) | 9 | 36,791,154 | 467,121.5 | 0.99 | 0.01 |
| Temperature annual range (BIO7) | 10 | 36,884,173 | 374,101.8 | 0.99 | 0.01 |
| Temperature annual range (BIO7) | 11 | 36,900,702 | 357,573.3 | 0.99 | 0.01 |
| Temperature annual range (BIO7) | 12 | 36,901,230 | 357,044.6 | 0.99 | 0.01 |
| Roughness | 8 | 1,748,254,398 | 943,622,810.2 | 0.65 | 0.35 |
| Roughness | 9 | 1,902,443,293 | 789,433,915.3 | 0.71 | 0.29 |
| Roughness | 10 | 2,003,998,547 | 687,878,661.5 | 0.74 | 0.26 |
| Roughness | 11 | 2,026,139,184 | 665,738,024.0 | 0.75 | 0.25 |
| Roughness | 12 | 2,026,755,618 | 665,121,590.2 | 0.75 | 0.25 |
| Minimum annual streamflow | 8 | 83,516,408 | 1,890,585,000.9 | 0.04 | 0.96 |
| Minimum annual streamflow | 9 | 169,709,841 | 1,804,391,568.5 | 0.09 | 0.91 |
| Minimum annual streamflow | 10 | 252,658,745 | 1,721,442,664.2 | 0.13 | 0.87 |
| Minimum annual streamflow | 11 | 290,452,985 | 1,683,648,424.4 | 0.15 | 0.85 |
| Minimum annual streamflow | 12 | 291,412,674 | 1,682,688,735.3 | 0.15 | 0.85 |

Supplementary figures


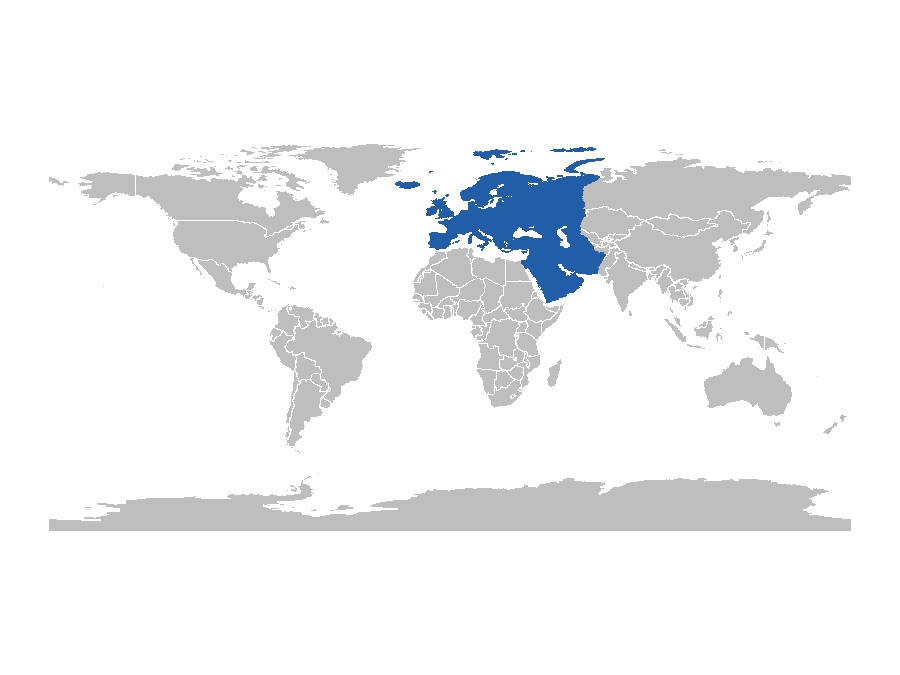


**Figure S1**: Study area (in blue).


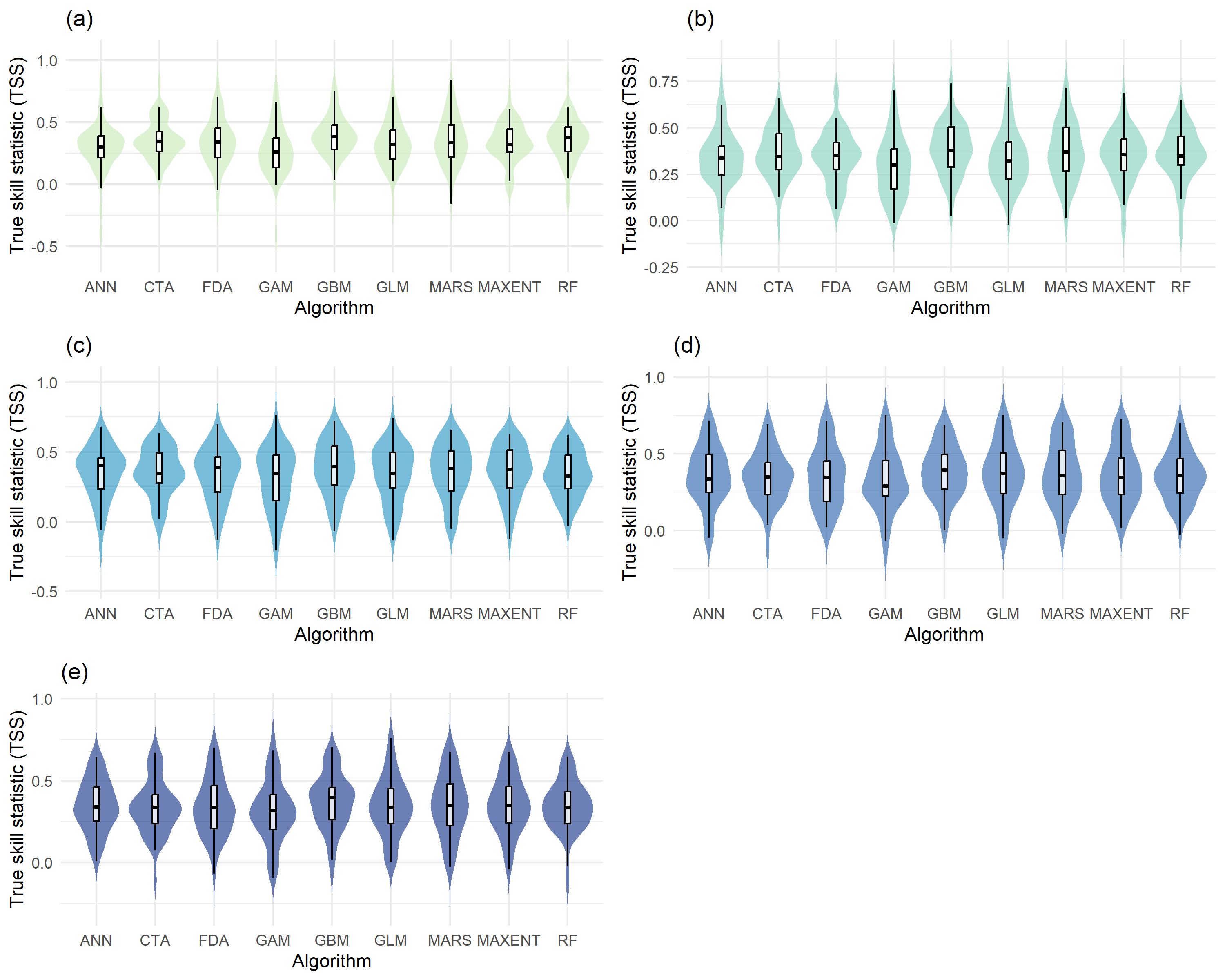


**Figure S2**: Predictive performance as true skill statistic (TSS) of algorithms used to employ species distribution models (SDMs) for 49 freshwater fish species across resolutions 8 (**a**), 9 (**b**), 10 (**c**), 11 (**d**), 12 (**e**).


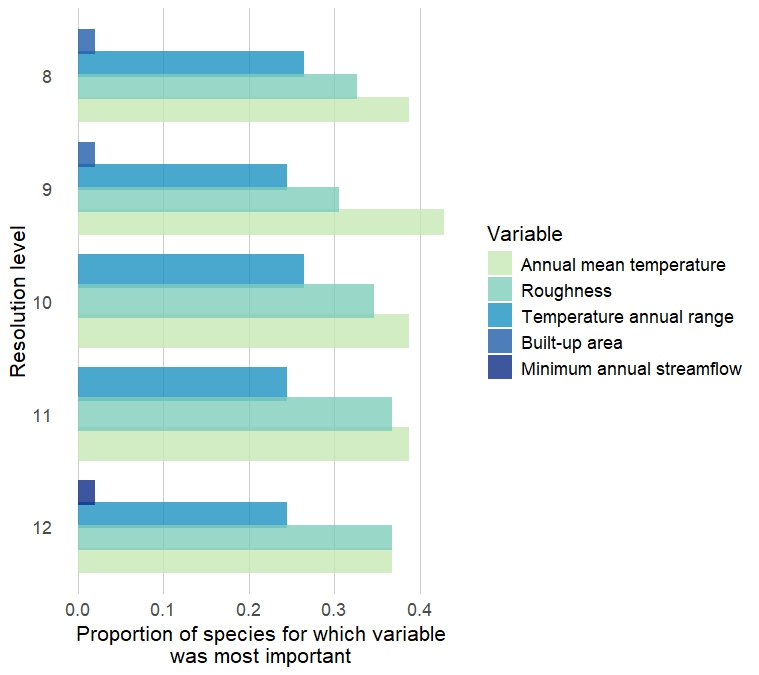


**Figure S3**: Proportion of freshwater fish species for which a given variable was the most important for each resolution level.

Supplementary text

To verify the extent to which the aggregation of predictor variables to hydrobasins units may have affected their heterogeneity and variance, we quantified the variability of values of the predictor variable within (intra-basin) and between (inter-basin) hydrobasins at each level. First, we calculated the sum of squares of variability within hydrobasins (SSW) based on the environmental variable values of the grid cells (i) in the hydrobasins (j) (SSW; Eq. S1).

$$SSW=\sum_{j}^{N} \sum_{i}^{M} \left( x_{i,j}-\overline{x_{j}} \right)^{2}$$

Then, we calculated the sum of squares of variability between hydrobasins (SSB; Eq. S2).

$$SSB=\sum_{j}^{N} N\left( \overline{x_{j}}-\overline{x} \right)^{2}$$

We calculated the sum of squares within and between hydrobasins for mean temperature (BIO1), temperature annual range (BIO7), topography, and minimum streamflow at each spatial resolution. We then compared which one of the two squared sums was dominant per each variable across resolutions.

Inter-basin sum of squares was dominant for mean temperature (BIO1), temperature annual range (BIO7), and topography across all the five resolutions, but it was not for minimum streamflow (**Table S9**). At higher resolutions, intra-basin sum of squares decreased for all variables, while inter-basin sum of squares increased.
